# Validation of extracellular ligand–receptor interactions by Flow-TriCEPS

**DOI:** 10.1186/s13104-018-3974-5

**Published:** 2018-12-05

**Authors:** Laura A. Lopez-Garcia, Levent Demiray, Sandra Ruch-Marder, Ann-Katrin Hopp, Michael O. Hottiger, Paul M. Helbling, Maria P. Pavlou

**Affiliations:** 1 Dualsystems Biotech A.G., Grabenstrasse 11a, 8952 Schlieren, Switzerland; 2 Department of Molecular Mechanisms of Disease, University of Zurich, 8057 Zurich, Switzerland; 3 Molecular Life Science PhD Program, The Life Science Zurich Graduate School, 8057 Zurich, Switzerland

**Keywords:** Flow-TriCEPS, HATRIC, LRC, Knock down, siRNA, Candidate verification

## Abstract

**Objective:**

The advent of ligand-based receptor capture methodologies, allows the identification of unknown receptor candidates for orphan extracellular ligands. However, further target validation can be tedious, laborious and time-consuming. Here, we present a methodology that provides a fast and cost-efficient alternative for candidate target verification on living cells.

**Results:**

In the described methodology a ligand of interest (e.g. transferrin, epidermal growth factor or insulin) was conjugated to a linker (TriCEPS) that carries a biotin. To confirm ligand/receptor interactions, the ligand–TriCEPS conjugates were first added onto living cells and cells were subsequently labeled with a streptavidin-fluorophore and analyzed by flow cytometry (thus referred as Flow-TriCEPS). Flow-TriCEPS was also used to validate identified receptor candidates when combined with a siRNA knock down approach (i.e. reduction of expression levels). This approach is versatile as it can be applied for different classes of ligands (proteins, peptides, antibodies) and different cell lines. Moreover, the method is time-efficient since it takes advantage of the large variety of commercially available (and certified) siRNAs.

**Electronic supplementary material:**

The online version of this article (10.1186/s13104-018-3974-5) contains supplementary material, which is available to authorized users.

## Introduction

Identifying the protein binding receptors of key ligands provides valuable mechanistic information regarding signal transduction, drug action or off-target effects. Recognizing the unmet need to decipher transient extracellular interactions, the ligand-based receptor capture (LRC) technology was developed [1, 2]. The advantages of the LRC-TriCEPS™ (and its latest development LRC-HATRIC [3]) methodology include versatility, no need for genetic manipulation, target identification on living cells and the ability to capture both stable and transient interactions.

The LRC methodologies have been applied to study numerous ligands ranging from small molecules to intact viruses and have revealed known and novel interactors [1, 3–8]. As with every discovery-based methodology, the resulting candidate targets should be further validated and confirmed, which can be a tedious, laborious and time-consuming exercise.

In the present technical note, we present a method for the fast validation and confirmation of receptor candidates, which was demonstrated using different ligands and their well-established targets.

## Main text

### Methods

#### TriCEPS-ligand coupling

20 μg of transferrin (TRFE), epidermal growth factor receptor (EGFR) antibody, insulin (INS) and glycine (Gly) were coupled to 10 μg of TriCEPS v.2.0 (biotin moiety) or TriCEPS-TAMRA (fluorophore moiety) in a total volume of 50 μl 25 mM HEPES pH 8.2 (90 min at 22 °C). Upon coupling, non-reacted *N*-hydroxysuccinimide (NHS) was quenched with 20 μg Gly.

#### Cell incubation with TriCEPS-coupled ligand

MDA-MB-231 and HEK293 cells were collected using 5 mM ethylene-diamine-tetra-acetic acid, washed once with phosphate-buffered saline (PBS) pH 6.5 and incubated with 1.2 μg of the corresponding TriCEPS-ligands (500,000 cells in 200 μl PBS pH 6.5 for 60 min at 4 °C). For competition experiments, cells were incubated with different amounts of unlabeled ligand TRFE or bovine serum albumin (BSA) (20 min at 22 °C) in PBS pH 7.4 prior to addition of the TriCEPS-coupled ligand.

#### Flow cytometry

Upon incubation of cells with TriCEPS–coupled ligands, cells were washed twice with 400 μl of ice cold PBS buffer pH 6.5, labelled with streptavidin-R-phycoerythrin (PE) (Jackson) (30 min at 4 °C in the dark), washed twice and analyzed by flow cytometry.

#### siRNA knock down of receptor candidates

Ten nanomolar of scramble siRNA (AllStars Negative Control, Qiagen) and siRNA against human transferrin receptor (TFR1) (Hs_TFRC_7, Hs_TFRC_5 and Hs_TFRC_11, Qiagen), human EGFR (Hs_EGFR_10, Qiagen), human insulin receptor (INSR) (Hs_INSR_3 and Hs_INSR_4, Qiagen) and human insulin-like growth factor receptor (IGF1R) (Hs_IGF1R_8 and Hs_IGF1R_6, Qiagen) were used for reverse transfection of MDA-MB-231 and HEK293 cells on a 6 well plate format using Lipofectamine RNAiMax (Invitrogen). The knock down efficiency of TFR1 on mRNA level in MDA-MB-231 cells was monitored by qRT-PCR. Knock down efficiency was tested by flow cytometric analysis using PE anti human CD71 (TFR1) (BioLegend), PE anti human EGFR (BioLegend), PE anti human CD220 (INSR) (BD Bioscience) and PE anti human CD221 (IGF1R) (BD Bioscience) antibodies using recommended conditions.

#### Microscopy using TriCEPS-TAMRA

HEK293 cells were plated on cover slips previously coated with Poly l-Lys (0.01%) (EMD Millipore) on 12 well plates and the ligand–TriCEPS–TAMRA conjugates (8 μg) were added in 500 μl RPMI media containing 1% fetal bovine serum (45 min at 4 °C, in the dark). Upon incubation, cells were fixed with 4% paraformaldehyde (15 min at 22 °C in the dark), and the cover slips were transferred to a microscope slide containing 30 μl 4,6-diamidino-2-phenylindole (DAPI) (Invitrogen). Imaging was performed using the Leica SP8 inverse confocal microscope with support of the Center for Microscopy and Image Analysis, University of Zurich.

### Results

#### Development of Flow-TriCEPS as a method to determine ligand binding on living cells

In the first step of a LRC experiment, the tri-functional molecule (TriCEPS or HATRIC) is conjugated to the primary amines of the ligand of interest via a NHS-ester (Additional file 1: Figure S1). During the incubation of tri-functional molecule–ligand conjugates with mildly oxidized cells the second moiety (hydrazone) reacts with aldehydes of cell surface glycoproteins and decorates the cell surface. Finally, the third moiety (i.e. biotin) is used in order to pull down and thus enrich the tagged proteins [1, 3]. When conjugates are added on non-oxidized cells, the hydrazone should not react (no aldehydes) and conjugates should bind only to the target of interest (if expressed). To confirm the binding of a ligand to its receptor, the third moiety (i.e. biotin) can be replaced or interact with a fluorophore and the binding visualized by microscopy or flow cytometry.

To explore this possibility, we used TRFE as ligand, because its target TFR1 (also known as CD71) is expressed in the vast majority of cell types including the breast cancer cell line (MDA-MB-231) [9]. TRFE coupled to TriCEPS (TRFE-TriCEPS) was added on oxidized and non-oxidized MDA-MB-231 cells. The TriCEPS molecule quenched with glycine to hydrolyze the NHS group was used as a negative control. Samples were labeled with streptavidin-PE (Str-PE) that reacts with biotin of TriCEPS and analyzed by flow cytometry. In parallel, the same number of cells was incubated with Str-PE to assess the background signal. As shown by the shift of median fluorescence values, both conjugates bound to the surface of oxidized cells to a comparable extent likely due to the covalent labeling of glycoproteins (Additional file 1: Figure S1, left panel). Non-oxidized cells incubated with quenched TriCEPS (Gly-TriCEPS) showed a strong reduction of the median fluorescence values compared to cells incubated with TRFE-TriCEPS (Additional file 1: Figure S1, right panel). Given that quenched TriCEPS should not react with non-oxidized cells, the observed shift in the median fluorescence intensity in the cells treated with TRFE-TriCEPS was very likely due to the binding of TRFE to its corresponding target on the surface of the cells.

**Fig. 1:**
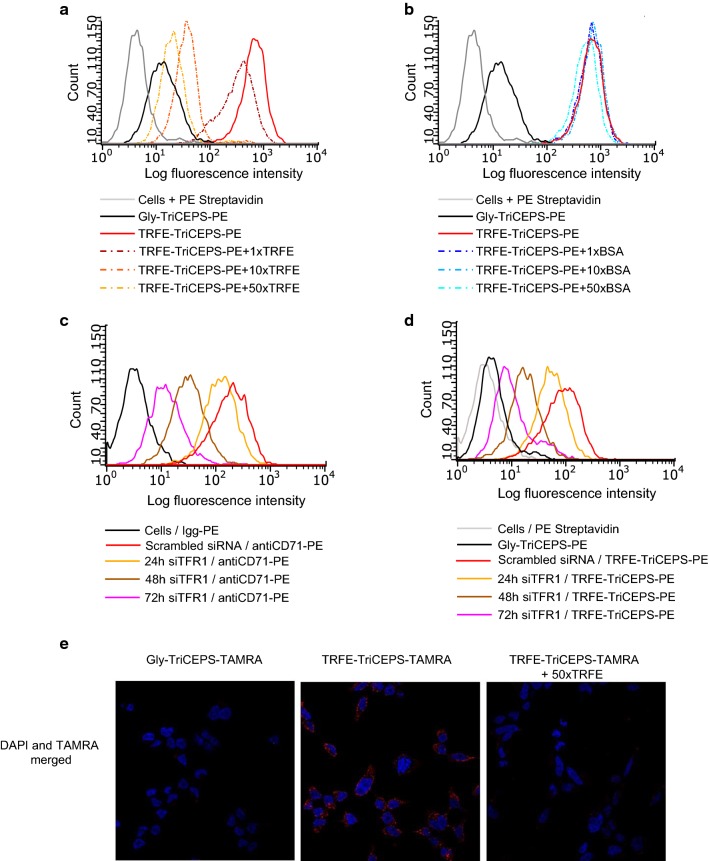
Development of Flow-TriCEPS as a method to determine ligand binding on living cells. **a** Flow cytometric analysis to determine binding of transferrin coupled to TriCEPS (TRFE-TriCEPS) to the surface of living MDA-MB-231 cells (red line). Competition assay by adding increasing amount of unlabeled TRFE. A representative experiment out of two is shown. **b** Addition of BSA, cannot compete the TRFE-TriCEPS-PE signal on MDA-MD-231 cells (red line). **c** Flow cytometric analysis to determine binding of antiCD71-PE labelled on MDA-MB-231 cells transfected with siRNA TFR1 (Hs_TFR1_5) at different time points, Igg-PE was used as control. **d** Flow cytometric analysis to determine binding of TRFE-TriCEPS on MDA-MB-231 cells transfected with siRNA TFR1 (Hs_TFR1_5) at different time points, cells labelled with PE-Streptavidin were used as control. Similar results were obtained by using Hs_TFR1_11 and Hs_TFR1_7 siRNA for 72 h. A representative experiment out of 3 is shown. **e** Confocal microscopy using TriCEPS-TAMRA to detect binding of TRFE-TriCEPS-TAMRA on the surface of living HEK293 cells. As control of the specificity TRFE-TriCEPS was out-competed with excess of unlabeled TRFE

To assess if the observed signal in the above-described TRFE-TriCEPS experiment originated from the specific interaction of TRFE with its target, a competition experiment was performed. Non-oxidized cells were pretreated with increasing amounts of unlabeled TRFE or BSA (1, 10 and 50 times excess) before they were incubated with the TRFE–TriCEPS conjugate. In parallel, one sample was treated with quenched TriCEPS (Gly-TriCEPS). All samples were subsequently labeled with Str-PE. As above, cells of one sample were incubated only with Str-PE to assess the background signal. As shown by the shift of median fluorescence values, the intensity decreased for the samples that were pretreated with unlabeled TRFE in a dose–response manner, compared to the non-pretreated cells (Fig. 1a). Pre-treatment of cells with increasing amounts of BSA did not result in a decrease in the signal intensity (Fig. 1b), indicating that the binding of the TRFE–TriCEPS conjugate was out-competed by the excess of unlabeled TRFE and that the observed competition was not due to interference caused by the high protein concentration in the sample, but rather specific.

To further validate that the signal was due to a specific interaction of TRFE with its receptor, the expression of TFR1 was downregulated in MDA-MB-231 cells (Additional file 2: Figure S2a). Cells were transfected with anti-TFR1 siRNA for 24, 48 or 72 h and the expression levels of TFR1 on the cell surface were quantified by incubating cells with an anti-CD71-PE antibody followed by flow cytometric analysis. Cells transfected with scramble siRNA served as negative control (Additional file 2: Figure S2b, left panel). Based on the observed shift of median fluorescence with the transfected cells (Fig. 1c, Additional file 2: Figure S2b), the TFR1 levels were over time gradually reduced, indicating that the used siRNA was indeed specific for TFR1. When the transfected cells were subsequently incubated with the TRFE–TriCEPS conjugate and labeled with Str-PE, the acquired fluorescence signal closely mimicked the signal intensity staining of the anti-CD71 antibody, suggesting that the observed signal was due to the interaction of TRFE with TFR1 (Fig. 1d).

To ensure that the ligand–TriCEPS conjugate did not enter the cells, cells treated in the same manner as described above were analyzed by microscopy (Fig. 1e). The TRFE–TriCEPS–TAMRA conjugate was only located on the surface of cells. Together, these experiments provide strong evidence that coupling of TriCEPS to TRFE followed by flow cytometry is suitable to monitor the binding of ligands on the surface of living cells.

#### Verification of ligand binding to LRC-HATRIC identified targets by siRNA knockdown of putative candidates and subsequently analysis with Flow-TriCEPS

We next investigated if the developed Flow-TriCEPS pipeline was also applicable to different classes of ligands. Two ligands were selected (i) an antibody against EGFR (anti-EGFR) and (ii) a peptide (insulin) that is known to bind to the INSR and IGF1R [10]. The targets of the above-mentioned ligands were confirmed by independent LRC-HATRIC experiments (Additional file 3: Figure S3).

##### Anti-EGFR antibody

**Fig. 2:**
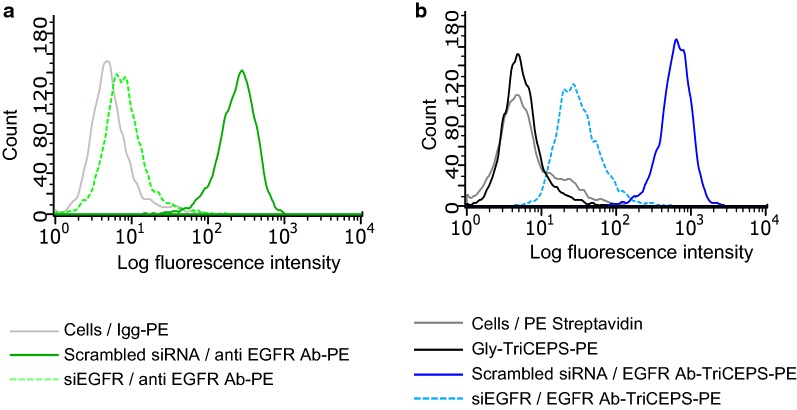
Flow-TriCEPS knock down target validation of a protein (TRFE) and an antibody (anti EGFR) on MDA-MB-231 cells. **a** Flow cytometric analysis comparing binding of anti EGFR-PE labelled antibody on MDA-MB-231 cells transfected with scrambled or EGFR siRNA (Hs_EGFR_10) for 72 h. Igg-PE was used as control. A representative experiment out of two is shown. **b** Flow cytometric analysis comparing binding of EGFR Ab-TriCEPS on MDA-MB-231 cells transfected with scrambled or EGFR siRNA (Hs_EGFR_10) for 72 h. Gly-TriCEPS and cells labelled with PE-streptavidin were used as control. A representative experiment out of two is shown

To verify that the anti-EGFR antibody would only bind to EGFR, we reduced the expression of EGFR by siRNA knockdown and assessed binding of the ligand in this perturbed conditions by Flow-TriCEPS. MDA-MB-231 cells were transfected for 72 h with anti-EGFR siRNA and scramble siRNA, respectively. Reduction of EGFR protein levels was assessed by incubating cells with an anti-EGFR-PE antibody followed by flow cytometric analysis. Based on the shift of median fluorescence, the expression level of EGFR on the cell surface was drastically reduced in the cells transfected with anti-EGFR siRNA compared to cell transfected with scrambled siRNA, confirming the knockdown of EGFR on protein level (Fig. 2a). When those transfected cells were incubated with an anti-EGFR-antibody–TriCEPS conjugate and further labeled with Str-PE, the acquired fluorescence signal was comparably reduced, indicating that cells with lower EGFR expression levels bound the anti-EGFR-antibody to lower extent (Fig. 2b).

##### Insulin

**Fig. 3:**
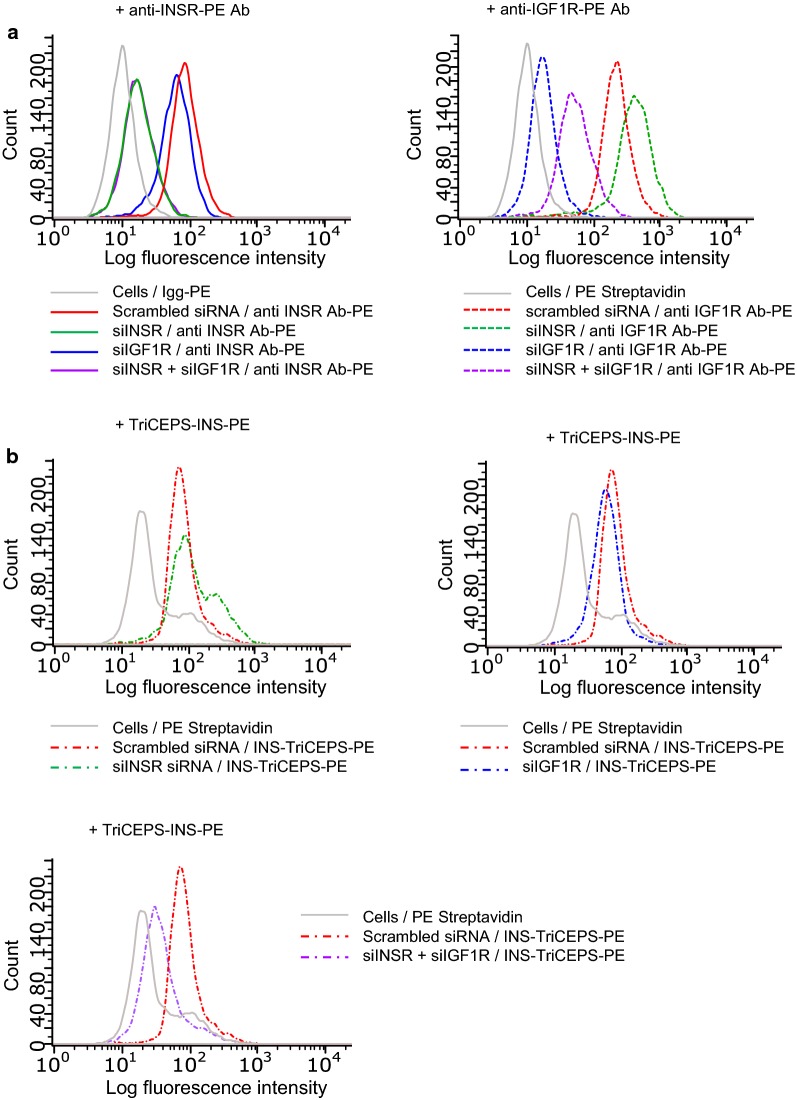
Flow-TriCEPS knock down target validation of a peptide hormone (insulin) on HEK293 cells. **a** Flow cytometric analysis to determine binding of anti INSR Ab-PE (left panel) and anti IGF1R Ab-PE (right panel) on HEK293 cells transfected with siRNA to knock down INSR (Hs_INSR_3), IGF1R (Hs_IGF1R_8) or both receptors simultaneously for 72 h. Cells transfected with scrambled siRNA were used as control. A representative experiment out of 3 is shown. Similar results were obtained by using Hs_INSR_4 and Hs_IGF1R_6 siRNA. **b** Flow cytometric analysis to determine binding of INS-TriCEPS on HEK293 cells transfected with siRNA to knock down INSR (Hs_INSR_3) for 72 h (top left panel), siRNA to knock down IGF1R (Hs_IGF1R_8) for 72 h (top right panel) and siRNA to simultaneously knock down INSR and IGF1R for 72 h (low panel). Cells transfected with scrambled siRNA were used as control. A representative experiment out of 3 is shown. Similar results were obtained by using Hs_INSR_4 and Hs_IGF1R_6 siRNA

To provide further evidence for the binding of INS to INSR and IGF1R, the expression levels of either INSR or IGF1R alone or in combination in HEK293 cells were perturbed. Therefore, cells were transfected with a siRNA against INSR and IGF1R individually or in combination. The same transfection of cells with scrambled siRNA served as negative control. The success of transfection was assessed by incubating transfected cells with PE-labeled anti-human CD220 (against INSR) and anti-human CD221 (against IGF1R) antibodies followed by flow cytometric analysis. Based on the shift of median fluorescence across cells transfected with the different siRNA combinations, the protein expression level of INSR was not affected in cells transfected with either scramble siRNA or anti-IGF1R siRNA. However, INSR protein levels were drastically reduced in cells transfected with only siRNA against INSR or double transfected cells (i.e. INSR and IGF1R) (Fig. 3a, left panel). In a similar manner, the protein level of IGF1R was not affected in cells transfected with scramble siRNA but was drastically reduced in cells transfected with a siRNA against IGF1R or doubly transfected cells (i.e. INSR and IGF1R) (Fig. 3a, right panel). Notably, protein levels of IGF1R showed a small increase in cells lacking INSR, an observation reported already before in the literature [10].

Finally, we investigated the binding of INS in transfected cells using Flow-TriCEPS. When compared to cells transfected with scrambled siRNA, the generated signal by INS-TriCEPS was not reduced in cells expressing neither INSR nor IGF1R (Fig. 3b, left and right top panels respectively). This could be due to functional compensation between INSR and IGF1R [10]. However, fluorescence signal is drastically reduced in cells lacking both, INSR and IGF1R (Fig. 3b, low panel), suggesting that combinatorial knock-down of multiple targets may be necessary to demonstrate reduced ligand binding.

### Discussion

Validating previously identified ligand–receptor interactions can for different reasons be challenging and time consuming. Here, we provide evidence in three different conditions that the developed Flow-TriCEPS methodology can monitor binding of extracellular ligands to their receptors on living cells. Additionally, we showed that candidate targets identified by the LRC methodologies can be quickly validated by combining Flow-TriCEPS with a siRNA mediated knock down approach for a particular receptor protein. This approach is versatile as it can be applied for different classes of ligands (proteins, peptides, antibodies) and different cell lines. The method is also time- and cost-efficient and takes advantage of the large variety of commercially available (and certified) siRNAs. Additionally, should a ligand bind to several interaction candidates, the combinatorial knock down of these targets can be easily performed as demonstrated in the case of INS.

## Limitations

- Not all cell types can be readily transfected, which might require additional optimization.
- In case that a ligand has more than one candidate targets, the combinatorial knock down of these should be performed, as single knock downs may lead to increase of the other targets compensating ligand binding.

## Additional files

**Additional file 1: Figure S1.** **a** Representation of HATRIC, TriCEPS v.2.0 and TriCEPS–TAMRA molecules. **b** TriCEPS–ligand conjugates bind to the cell surface on mildly oxidized MDA-MB-231 cells to the same extend (left panel). TriCEPS coupled ligand binds only to the target receptor on non-oxidized cells (right panel). A representative experiment out of 3 is shown.

**Additional file 2: Figure S2.** **a** qRT-PCR confirming knock down of TFR1 (Hs_TFR1_5) at RNA level on MDA-MB-231 cells at different time points. **b** Igg-PE was used as control at different TFR1 knock down time points. Igg-PE signal is overlapping and displaying no fluorescent shift (right panel).

**Additional file 3: Figure S3.** LRC- HATRIC experiment comparing TRFE-HATRIC and EGFR Ab-HATRIC samples on MDA-MB-231 cells (left panel) and INS-HATRIC and TRFE-HATRIC samples on HEK293 cells (right panel). Data is shown at the protein level and proteins were annotated using the Uniprot database. Y axis = − Log10 (adj. *p* value), X-axis = log_2_ fold change compared to the other sample.

## Abbreviations

LRC: ligand-based receptor capture
TRFE: transferrin
EGFR: epidermal growth factor receptor
INS: insulin
Gly: glycine
NHS: *N*-hydroxysuccinimide
BSA: bovine serum albumin
PE: R-Phycoerythrin
TFR1: transferrin receptor
INSR: insulin receptor
IGFR1: insulin-like growth factor receptor
Str-PE: streptavidin-PE
