# Supplementary material for: Validation of extracellular ligand–receptor interactions by Flow-TriCEPS
Source: BMC Res Notes. 2018 Dec 5;11:863. doi: 10.1186/s13104-018-3974-5 (PMC6280402; doi:10.1186/s13104-018-3974-5)
Supplement: Supplementary file 1 — Additional file 1: Figure S1. a Representation of HATRIC, TriCEPS v.2.0 and TriCEPS–TAMRA molecules. b TriCEPS–ligand conjugates bind to the cell surface on mildly oxidized MDA-MB-231 cells to the same extend (left panel). TriCEPS coupled ligand binds only to the target receptor on non-oxidized cells (right panel). A representative experiment out of 3 is shown. [file 13104_2018_3974_MOESM1_ESM.pptx]

## Slide 1
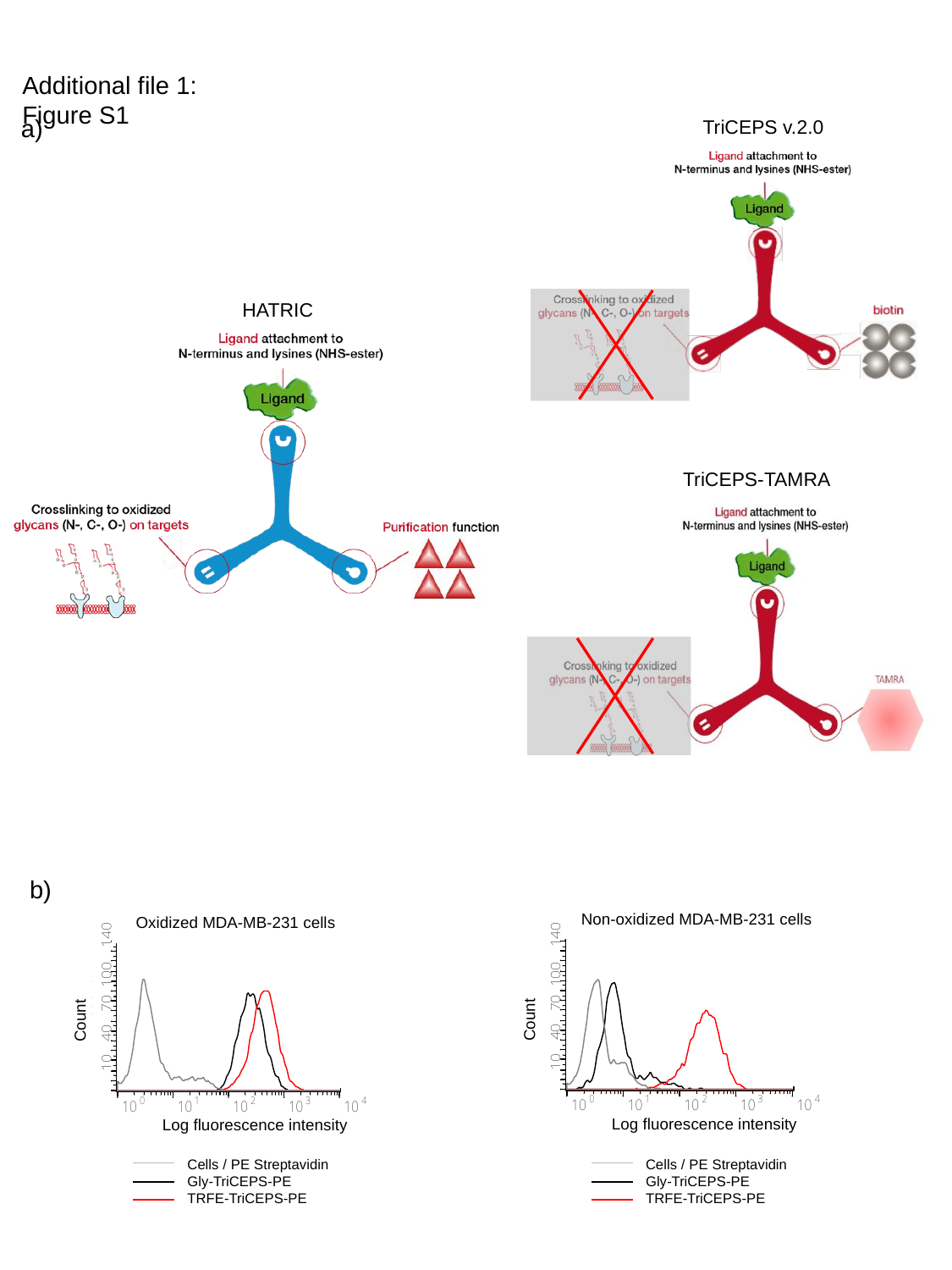

Additional file 1: Figure S1
a)
TriCEPS v.2.0
HATRIC
TriCEPS-TAMRA
b)
Non-oxidized MDA-MB-231 cells
Oxidized MDA-MB-231 cells
Count
Count
Log fluorescence intensity
Log fluorescence intensity
Cells / PE Streptavidin
Gly-TriCEPS-PE
TRFE-TriCEPS-PE
Cells / PE Streptavidin
Gly-TriCEPS-PE
TRFE-TriCEPS-PE
