# Supplementary material for: Validation of extracellular ligand–receptor interactions by Flow-TriCEPS
Source: BMC Res Notes. 2018 Dec 5;11:863. doi: 10.1186/s13104-018-3974-5 (PMC6280402; doi:10.1186/s13104-018-3974-5)
Supplement: Supplementary file 2 — Additional file 2: Figure S2. a qRT-PCR confirming knock down of TFR1 (Hs_TFR1_5) at RNA level on MDA-MB-231 cells at different time points. b Igg-PE was used as control at different TFR1 knock down time points. Igg-PE signal is overlapping and displaying no fluorescent shift (right panel). [file 13104_2018_3974_MOESM2_ESM.pptx]

## Slide 1
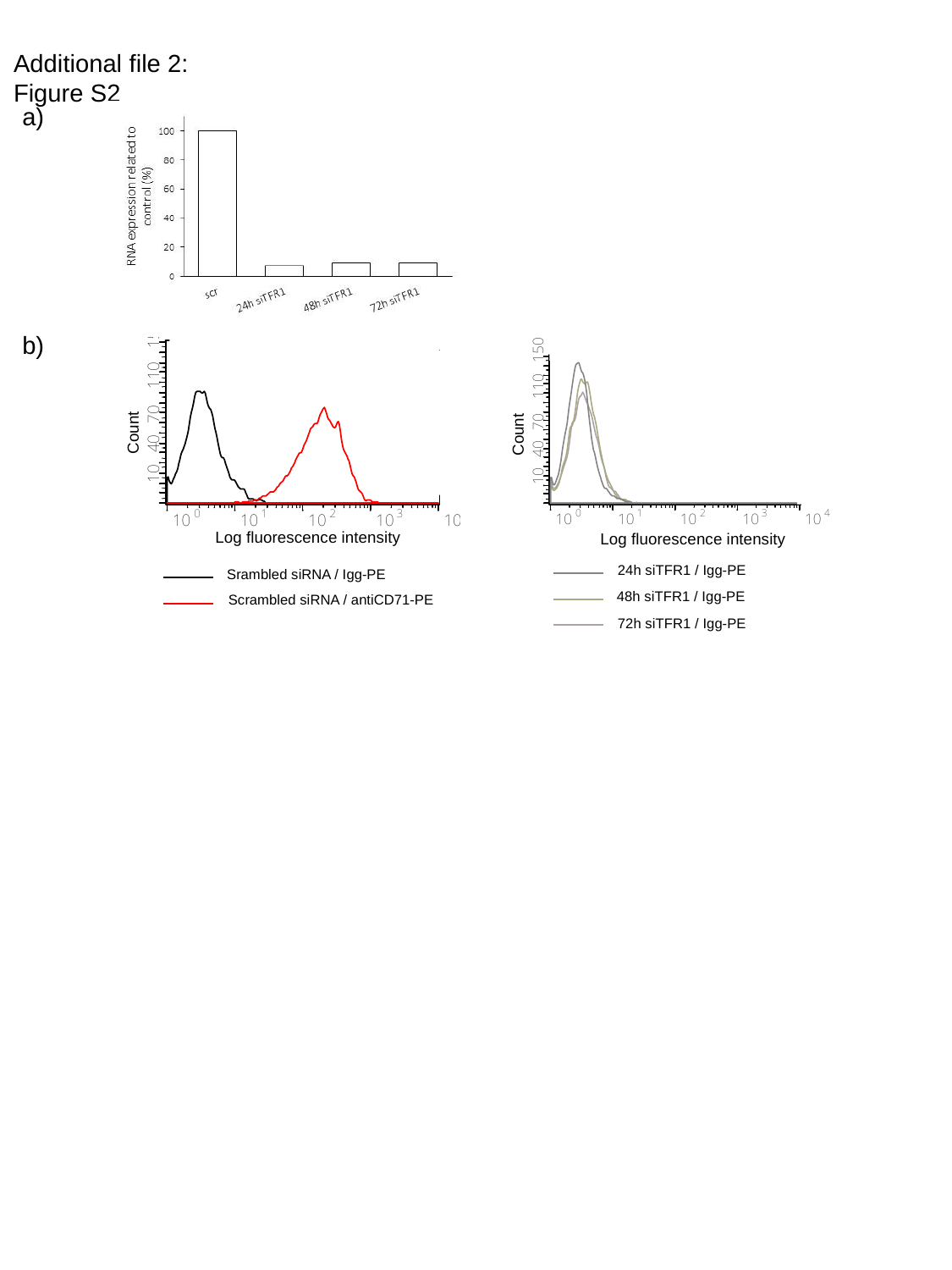

Additional file 2: Figure S2
a)
b)
Count
Count
Log fluorescence intensity
Log fluorescence intensity
24h siTFR1 / Igg-PE
48h siTFR1 / Igg-PE
72h siTFR1 / Igg-PE
Srambled siRNA / Igg-PE
Scrambled siRNA / antiCD71-PE
