# Supplementary material for: Validation of extracellular ligand–receptor interactions by Flow-TriCEPS
Source: BMC Res Notes. 2018 Dec 5;11:863. doi: 10.1186/s13104-018-3974-5 (PMC6280402; doi:10.1186/s13104-018-3974-5)
Supplement: Supplementary file 3 — Additional file 3: Figure S3. LRC- HATRIC experiment comparing TRFE-HATRIC and EGFR Ab-HATRIC samples on MDA-MB-231 cells (left panel) and INS-HATRIC and TRFE-HATRIC samples on HEK293 cells (right panel). Data is shown at the protein level and proteins were annotated using the Uniprot database. Y axis = − Log10 (adj. p value), X-axis = log2 fold change compared to the other sample. [file 13104_2018_3974_MOESM3_ESM.pptx]

## Slide 1
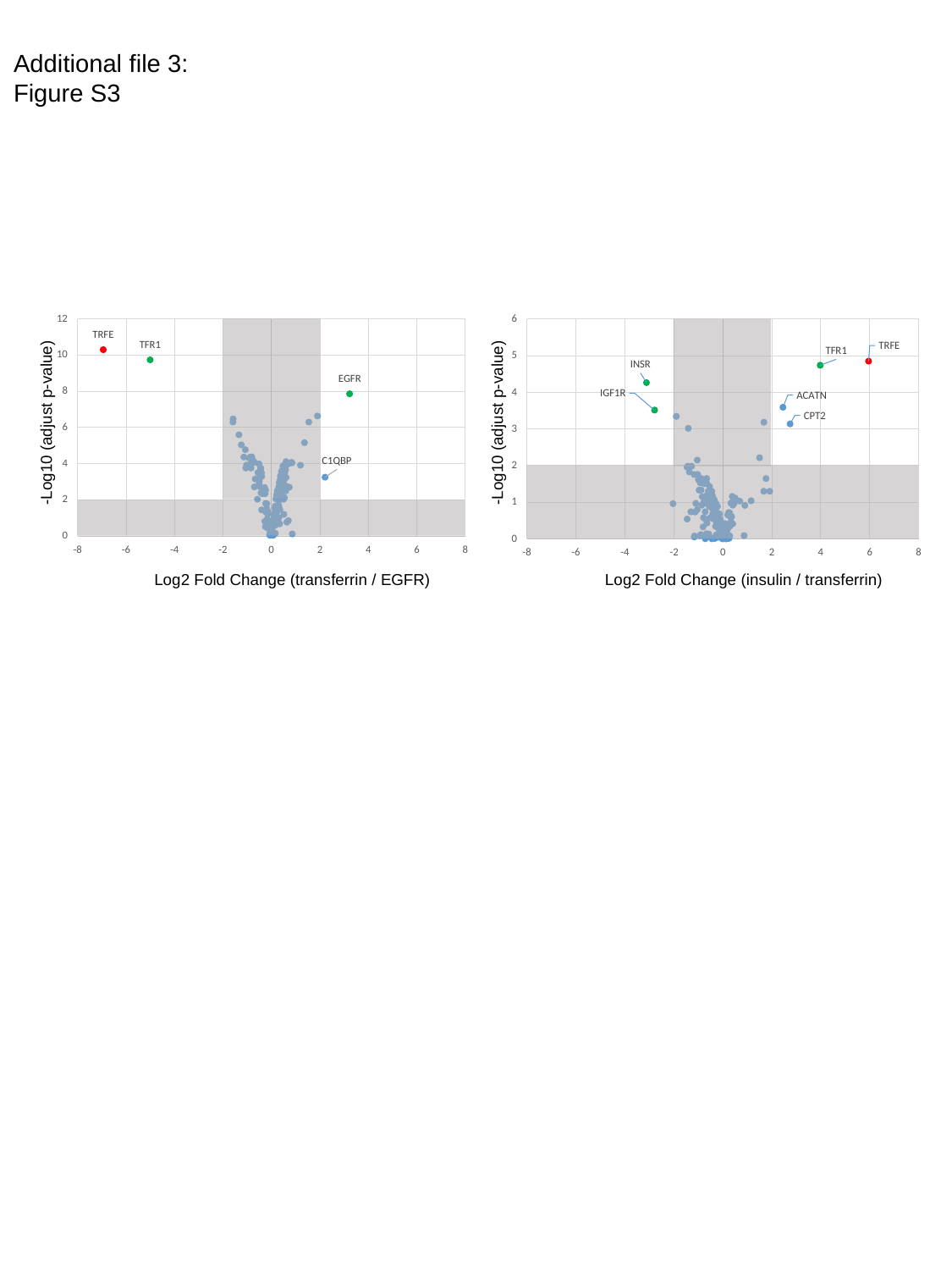

Additional file 3: Figure S3
-Log10 (adjust p-value)
-Log10 (adjust p-value)
Log2 Fold Change (transferrin / EGFR)
Log2 Fold Change (insulin / transferrin)
